# A Home-Based Individual Cognitive Stimulation Program for Older Adults With Cognitive Impairment: A Randomized Controlled Trial

**DOI:** 10.3389/fpsyg.2021.741955

**Published:** 2021-11-22

**Authors:** Rosa Silva, Elzbieta Bobrowicz-Campos, Paulo Santos-Costa, Ana Rita Cruz, João Apóstolo

**Affiliations:** ^1^Health Sciences Research Unit: Nursing, Nursing School of Coimbra, Coimbra, Portugal; ^2^Faculty of Psychology and Educational Sciences, Centre of 20th Century Interdisciplinary Studies, University of Coimbra, Coimbra, Portugal; ^3^HEI-Lab, Lusófona University, Lisbon, Portugal

**Keywords:** older adults, dementia, mild cognitive impairment, neurocognitive disorder, individual cognitive stimulation, caregiver

## Abstract

**Objective:** This study aims to assess the feasibility and meaningfulness of a home-based individual cognitive stimulation (iCS) program delivered by caregivers to persons with cognitive impairment (PwCIs). It also aims to assess whether the older adults receiving this program improved their cognitive, neuropsychiatric, and depressive symptoms and quality of life and whether their caregivers improved their mental and physical health.

**Methods:** A randomized controlled trial (RCT) was conducted with PwCI-caregiver dyads recruited from the community. Participants were allocated to two groups: intervention (*n* = 28) and control (*n* = 24). The intervention group received the European Portuguese version of the Individual Cognitive Stimulation Program—Making a Difference 3 (MD3-P). The control group received usual care. The iCS therapy program was implemented three times a week for 12 weeks. Caregivers were supported by the researchers to deliver the sessions at home. Participants were assessed at baseline and at the end of the intervention (week 13). Feasibility and meaningfulness were assessed through the attrition rate, adherence, and degree of satisfaction with the sessions. Four interviews were conducted (after week 13) to understand participants’ experiences.

**Results:** The attrition rate was 23.1%. The dyads reported that they did not have high expectations about the iCS program before starting the study. Nevertheless, as the program evolved, caregivers noted that their family members had improved some areas of functioning. Intention-to-treat analysis based on group differences revealed a significant improvement in PwCIs’ cognition, specifically in their orientation and ability to follow commands. The intervention had no impact on other variables such as caregivers’ physical and mental health.

**Conclusion:** The iCS program implemented by caregivers showed promising results in improving PwCIs’ cognition. The participants who completed the intervention attributed a positive meaning to the MD3-P, confirming it as a valid non-pharmacological therapeutic approach to reducing frailty in PwCIs in community settings.

**Clinical Trial Registration:** www.ClinicalTrials.gov, identifier [NCT03514095].

## Introduction

Providing conditions that promote healthy aging in community settings is a social priority. Aging is associated with increased prevalence of cognitive disorders, such as mild cognitive impairment (MCI) or dementia, also known as major neurocognitive disorders (American Psychiatric Association [APA], 2013; Prince et al., 2015; Apóstolo et al., 2016; Livingston et al., 2017). Several factors influence the onset and progression of late-life cognitive disorders, namely demographic, genetic, cardiovascular, behavioral, and psychosocial factors (Apóstolo et al., 2016). A deeper understanding of these factors is crucial for creating and activating mechanisms to prevent and treat MCI and reduce the prevalence of its more evolved forms. However, effective disorders-modifying therapies are still lacking. Recent studies (Panza et al., 2015, 2017) have shown that the adverse effects of late-life cognitive disorders can be prevented or minimized through the successful management of other age-related conditions, such as frailty.

Frailty is a clinical state resulting from aging-associated physiological and biological declines. It is characterized by a decrease of the individual’s homeostatic reserves, leading to diminished resistance to external agents and/or stressful events (Fried et al., 2001; Varadhan et al., 2008; Lang et al., 2009). Frailty consists of potentially reversible changes at different levels of individual functioning. According to the phenotypic model of frailty (Fried et al., 2001, 2004), these changes include (i) impaired mobility, strength, balance, and/or endurance, (ii) weight loss or undernutrition, and/or (iii) decrease in physical activity, representing a risk factor for adverse health outcomes such as falls, fractures, disability, dependency, hospitalization, institutionalization, or even death.

In alternative approaches, frailty is a multidimensional syndrome that can be measured by counting the number of health-related deficits, such as chronic comorbid and disabling illnesses, geriatric conditions, and disabilities (Deficit Accumulation Approach, Rockwood et al., 2005; Rockwood et al., 2007; Lacas and Rockwood, 2012) or examining the losses experienced in physical, psychological (mood and cognition), and social domains in combination with the effects of life-course determinants and multimorbidity (Integral Model of Frailty, Gobbens et al., 2010). Frailty-related cognitive losses can affect memory (Ruan et al., 2015), executive functions (Langlois et al., 2012; Robertson et al., 2013; Ruan et al., 2015), attention (Robertson et al., 2013; Ruan et al., 2015), language, visuospatial functions (Ruan et al., 2015), and processing speed (Langlois et al., 2012). However, in less severe cases, these losses may not be detected during cognitive screening due to older adults’ compensatory efforts (Ruan et al., 2015).

In a recent study, physical frailty was associated with late-life cognitive disorders (Panza et al., 2015). On the other hand, cognitive impairment increases the risk of physical frailty, which suggests that both conditions can influence each other (Robertson et al., 2013). Indeed, it is not uncommon for physical frailty to coexist with changes in cognition, which, upon reaching a certain level of clinical significance, point to the existence of cognitive frailty (Kelaiditi et al., 2013). Cognitive frailty is reversible if cognitive impairment is pre-MCI subjective cognitive decline and potentially reversible if cognitive impairment reaches the MCI level (Panza et al., 2015).

In line with this idea, a recent systematic review on the effectiveness of interventions in preventing pre-frailty and frailty progression in older adults showed that cognitive impairment is a major risk factor for vulnerability (Apóstolo et al., 2018). Studies have shown the benefits of a proximity multimodal care approach (both cognitive and physical activities) in preventing frailty (Panza et al., 2015). MCI is sensitive to a set of interventions that can slow down its progression into a potentially irreversible state, such as dementia (Apóstolo et al., 2016). Therefore, older adults with cognitive impairment require continuous care to meet their needs, delay the progression of frailty (Orrell et al., 2012a; Yates et al., 2014; Apóstolo et al., 2018), and increase their potential for self-care, autonomy, and independence (Milders et al., 2013).

Systematic reviews have shown that non-pharmacological interventions are an effective therapeutic option for maintaining cognitive performance, controlling neuropsychiatric symptoms (NPS), and improving quality of life (Olazarán et al., 2010; Woods et al., 2012; Silva et al., 2018, 2020). These interventions include reminiscence, training, cognitive stimulation, rehabilitation, and multisensory stimulation (Olazarán et al., 2010; Woods et al., 2012; Silva et al., 2018, 2020). Most of these therapeutic approaches are effective and can be used in conjunction with pharmacological treatments (Spector et al., 2008; Olazarán et al., 2010). Cognitive stimulation (CS) is a psychosocial approach that focuses on intellectual and social stimulation through relevant interaction activities and discussions in group or individual sessions (Spector et al., 2008; Woods et al., 2012; Apóstolo et al., 2014a). However, individual CS (iCS) has been underexplored (Quayhagen et al., 2000; Milders et al., 2013; Silva et al., 2020).

This individual approach can be developed at home at reasonable cost, and constitutes an innovative instrument in the context of *aging in place*. The development of caregiver-led iCS programs has attracted increasing research interest (Zientz et al., 2007; Yates et al., 2014, 2015a; Orgeta et al., 2015). Previous studies have shown that this therapeutic option is easy to apply and adapt to other settings besides the home environment (Quayhagen et al., 2000; Orrell et al., 2012a). Furthermore, iCS programs represent an alternative therapeutic approach in cases of impaired mobility or limited access to group CS programs (Orrell et al., 2012b). They are designed to be partially or fully delivered by the caregivers, who receive training, guidance, or supervision from a healthcare professional (Milders et al., 2013; Silva et al., 2020).

Caregivers can be spouses, family members, or friends interested in implementing the intervention (Quayagen et al., 1995; Milders et al., 2013; Aguirre et al., 2014; Yates et al., 2014, 2015a,b). A recent systematic review (Silva et al., 2020) found that caregiver-led individual cognitive intervention programs, including the iCS program, have improved cognitive performance, including immediate memory, attention, and problem-solving ability. Other authors have also reported that individual cognitive interventions have helped delay the institutionalization of persons with cognitive impairment (PwCIs; Moniz-Cook et al., 1998; Zientz et al., 2007; Orrell et al., 2012b).

In a systematic review, Silva et al. (2020) found few iCS programs in the literature, with the Making a Difference 3 (MD3) Individual Cognitive Stimulation Therapy being one of the most structured programs. The development of MD3 followed the guidance of the Medical Research Council framework for developing complex interventions and was funded by the United Kingdom’s National Institute of Health Research—Health Technology Assessment Program (Orrell et al., 2012b; Yates et al., 2014, 2015a,^2016^; Orgeta et al., 2015; Yates, 2016).

During a 25-week administration of MD3, Orgeta et al. (2015) found that people with dementia in the iCS group experienced better communication and relationship quality with their caregivers. Compared to the usual care (UC) group, caregivers in the iCS group also improved their health-related quality of life and had fewer depressive symptoms as they completed more MD3 sessions. However, other outcomes such as cognition, NPS, and quality of life were not statistically significant.

Therefore, further studies are needed to assess the impact of iCS, particularly the MD3 program, on PwCIs and their caregivers in different settings and cultures.

## Objective

This study aims to assess the effectiveness of the European Portuguese version of the MD3 (MD3-P) in improving the cognition (and its domains), quality of life, and neuropsychiatric and depressive symptoms of PwCIs. It also aims to compare the mental and physical health of caregivers of older adults who participated in the iCS activities to that of those who received UC. Finally, it aims to assess the feasibility and meaningfulness attributed to the MD3-P by PwCIs and their caregivers.

## Materials and Methods

This randomized controlled trial (RCT) had two arms: a UC group and a MD3-P group. There were two moments of blind assessment: at baseline (pre-intervention) and post-intervention at week 13 (outcome assessors were unaware of participant allocation). This study was approved by the Ethics Committee of the Regional Health Administration of Northern Portugal (number 27/2017). All ethical principles were observed in this study. All dyads (PwCI and caregiver) contacted by the research team were informed about the study’s objectives, the methodology, and the voluntary nature of their participation. They were also informed that they could withdraw at any time and that this decision would not affect the care being provided by the local healthcare units. All participants signed an informed consent form.

This RCT was registered on clinicaltrial.gov (record NCT03514095).

### Procedures

Participants were recruited in primary healthcare units of the Regional Health Administration of Northern Portugal. Before the study began, 11 formal meetings were held: four with the management team and seven with local clinicians (primary care nurses and general practitioners). The meetings aimed to prepare the team to refer the dyads. These professionals were explained the purpose of the study, including the MD3-P program and the RCT design, and the referral criteria.

First, the healthcare professionals selected potential participants and explained the study’s main objective to at least one member of the dyad. If the dyad showed interest in participating in the study, the healthcare professionals obtained their consent to be subsequently contacted by the research team. Then, a research team member met the dyad and screened both members for eligibility using inclusion/exclusion criteria. If the dyad met the inclusion criteria, they were given more information on the study and asked to read and sign a formal consent form. They were explained that they would be allocated to different groups and that if they were allocated to the control group, they could benefit from the intervention after study completion.

#### Inclusion Criteria

All participants met the following inclusion criteria:

(a) Aged 60 years or older;
(b) Diagnosed with MCI or dementia by a neurologist, psychiatrist, or general practitioner. If diagnosed by a general practitioner, the presence of diagnostic criteria as defined by the International Working Group on Mild Cognitive Impairment (Portet, 2006), the Diagnostic and Statistical Manual of Mental Disorders—Fourth or Fifth edition, or the ICD-9/10 (World Health Organization [WHO], 1977, 2004) was required (American Psychiatric Association [APA], 1994, 2002, 2013);
(c) Scored 2–20 points on the 6-item Cognitive Impairment Test (6-CIT; Brooke and Bullock, 1999; Portuguese version by Apóstolo et al., 2017);
(d) Were able to communicate effectively with others;
(e) Had no physical illness or disability affecting their participation;
(f) Lived in a community setting, either at their own home or in a family member’s home, and should not attend an adult day care center or any other institution of the same nature, such as a cognitive rehabilitation center/occupational therapy center;
(g) Had a caregiver (informal or formal) who completed, at least, primary school (1st–4th grade), available and willing to deliver the MD3-P program.

#### Exclusion Criteria

The following exclusion criteria were applied:

(a) Older adult/caregiver with a history of severe psychiatric illness, diagnosed before the age of 60; (b) Caregiver with cognitive impairment, even if a mild Neurocognitive Disorder according to DSM-5 criteria (American Psychiatric Association [APA], 2013), assessed by healthcare professionals when selecting potential participants.

### Randomization and Stratification

Data were collected at the participants’ homes. It included two moments of blind assessment: (i) At baseline, after inclusion in the study and before the randomization process (week 0, carried out by RS—member of the research team—and a psychologist hired for this task); (ii) 13 weeks after the intervention (week 13, carried out by ARC—member of the research team -, and another psychologist hired for this task).

Stratified randomization was performed by one member of the research team (PSC), who was blinded to the dyads. The variables for the stratification process were the PwCI’s sex and degree of cognitive impairment (mild or moderate, assessed using the 6-CIT). Participants were randomized using a randomization website.^1^

### Data Collection

The assessment tools administered at weeks 0 and 13 are presented below.

#### Assessment Tools

– Sociodemographic questionnaire developed by the research team to collect information on the PwCIs and caregivers, such as: age, sex, education level, type of relationship between dyad members, and marital status.

#### Primary Outcomes for the Persons With Cognitive Impairment

– Cognition: the Alzheimer’s Disease Assessment Scale (ADAS-Cog) by Rosen et al. (1984), European Portuguese version by Guerreiro et al. (2008). The ADAS-Cog comprises 11 tasks that evaluate the severity of cognitive and non-cognitive behavioral dysfunctions such as those related to memory, language, praxis, constructional praxis, and orientation. The higher the score, the greater the severity of cognitive impairment.
– Quality of life: the Quality of Life Scale—Alzheimer’s Disease (QoL-AD) by Logsdon et al. (1999), European Portuguese version by Bárrios (2012). This 13-item measure focuses on the different domains of patients’ lives, combining the information reported by them and their caregivers. The life domains assessed by the QoL-AD include physical condition, mood, interpersonal relationships, ability to participate in meaningful activities, financial situation, and overall perception of self and quality of life.

#### Secondary Outcomes for the Persons With Cognitive Impairment

– Neuropsychiatric symptoms (NPS): the Neuropsychiatric Inventory by Cummings et al. (1994), European Portuguese version by Leitão and Nina (2008). It was designed to assess the frequency and severity of behavioral disturbances in older adults with major neurocognitive disorders, such as delusions, hallucinations, dysphoria, anxiety, agitation/aggression, euphoria, disinhibition, irritability/lability, apathy, and aberrant motor activity.
– Depressive symptoms: the Geriatric Depression Scale (GDS-15) by Yesavage and Sheikh (1986), European Portuguese version by Apóstolo et al. (2014b). This brief self-report measure, developed from the GDS-30, evaluates the presence of depressive symptoms in older adults in the last 2 weeks. The higher the score, the higher the severity of symptoms.
– Quality of the Relationship between dyad members: the Quality of the Carer–Patient Relationship (QCPR) scale—PwCI version by Spruytte (2016), European Portuguese version by Silva et al. (2021). The QCPR scale includes two equal versions, one for PwCIs and another for caregivers. Each version assesses two dimensions of the emotion expressed: warmth/affection, the positive dimension; and conflict/criticism, the negative dimension. The total score ranged from 14 to 70 points. A score over 56 indicates a good quality relationship, a score between 56 and 42 indicates that the relationship is common, that is, a standard relationship, and a score under 42 reflects a poor quality relationship (Spruytte, 2016; Silva et al., 2021).

#### Outcomes for Caregivers

– Health status: the 12-Item Short Form Health Survey (SF-12) by Ware et al. (1995), European Portuguese version by Pais-Ribeiro (2005). The SF-12 is a self-reported measure assessing physical and mental health.
– Quality of the Relationship between dyad members: the QCPR scale—carer version by Spruytte (2016), European Portuguese version by Silva et al. (2021). For more details, see the QCPR—PwCI version described above.

### Intervention and Control Groups

Participants were randomly assigned to one of two arms: (i) intervention group receiving the iCS program MD3-P; (ii) control group receiving UC. Caregivers delivered the MD3-P sessions at home, three times, a week over 12 weeks. Concerning the UC group, participants maintained their usual activities at home or in other social/leisure settings, and no additional intervention was provided. Participants in both groups were asked to report all changes to the activity plan during the 12-week follow-up. None of the participants could be engaged in additional mentally stimulating activities (e.g., none of them attended a cognitive rehabilitation center, occupational therapy center, or adult day care center).

#### Intervention

The MD3 was translated, adapted, and validated for the Portuguese language and culture (Apóstolo et al., 2019; Silva, 2019). This iCS program is designed to be applied individually, three times a week, in 30-min sessions. The MD3 manual is divided into two parts. The first part of the manual teaches the caregiver how to use the manual and puts forward 13 principles for implementing the iCS program. The second part corresponds to the iCS sessions (Yates et al., 2015a; Apóstolo et al., 2019).

#### Caregiver Training

Caregiver training was developed in two moments. In the first moment, (a) a research team member delivered a 60-min theoretical-practical training session; (b) the dyad member received the MD3-P manual; (c) the caregiver was asked to read the 13 key principles and clarify any doubts with the research team.

In the second moment, a research team member was present during the first session of the MD3-P program delivered by the caregiver to assess their ability to implement the intervention, using a checklist with items reflecting the 13 key principles. The caregiver was considered fit to deliver the intervention if the session had run smoothly and followed more than seven of the 13 key principles. If the caregiver was unable to deliver the intervention, another theoretical-practical training session was scheduled between the caregiver and the research team.

#### Dyad Monitoring

During the 12 weeks, the dyads in the MD3-P group were contacted twice a week by telephone or in-person. This follow-up aimed to collect information on the number of completed sessions, average time per session, and difficulties experienced by the caregiver, provide support, and introduce complementary strategies. The research team also monitored the UC group through monthly telephone calls. This activity aimed to maintain contact with the dyads and record any changes in their dynamics.

#### Feasibility and Meaningfulness of the Individual Cognitive Stimulation Program

During the study, caregivers were asked to record each session’s details (e.g., time spent preparing the session, topic covered in the session, interaction during the session) to monitor the acceptability and applicability of the MD3-P program. Caregivers recorded their level of satisfaction using parameters such as the PwCIs’ ability to perform the tasks, the clarity of the instructions, or the session’s overall level of difficulty.

The attrition rate and the dyad’s adherence to the sessions were also analyzed. Finally, four interviews were conducted (after week 13) to explore the meanings attributed to the MD3-P program and understand the participants’ perceptions of the acceptability and applicability of the program. The following questions were asked: Do you think your involvement in this program was important? And if so, why? What did you like the most, and the least about the sessions? How did you benefit from this experience?

Participants who demonstrated involvement in the program were chosen to participate in the interview, in a total of two PwCIs and two caregivers.

## Analysis of Results

The significance level (*p*-value) was set at 5% for inferential analysis. Mann-Whitney *U*-test and Chi-Square test were used to compare the distribution of continuous and categorical variables between groups, respectively. The overall attrition rate (outcomes data in analysis/number of participants randomized) was calculated. Data on participants who dropped out of the study were subjected to intention-to-treat (ITT) analysis. Thus, all eligible individuals who received at least one session of iCS were included.

To determine the effect of the intervention, the pre- and post-intervention mean differences were calculated, as well as a non-standardized estimate (magnitude) of the intended outcomes. Thus, in addition to the significance level (*p*-value), the effect size (Cohen’s d) was considered. The statistical measure of the effect size (ES) Cohen’s d was calculated as a measure of effect size (ES) using the Z score resulting from the Mann-Whitney *U*-test, with the support of an online ES calculator for non-parametric tests (available at www.psychometrica.de/effect_size.html). The following formula was used: r = Z/√N. Subsequently, *r*-values were transformed to Cohen’s d at: https://www.psychometrica.de/effect_size.html#transform (Transformation of the ES d, r, f, Odds Ratio, and η^2^). The numbers needed to treat (NNT) were calculated based on the tables that support the conversion of Cohen’s d into these parameters (Santo and Daniel, 2017). Data were analyzed using IBM SPSS Statistics software (version 24, IBM SPSS, New York).

Following Bardin’s content analysis approach (Bardin, 2004), the qualitative data from the interviews were analyzed based on the categories established by combining inductive and deductive approaches.

## Results

**FIGURE 1:**
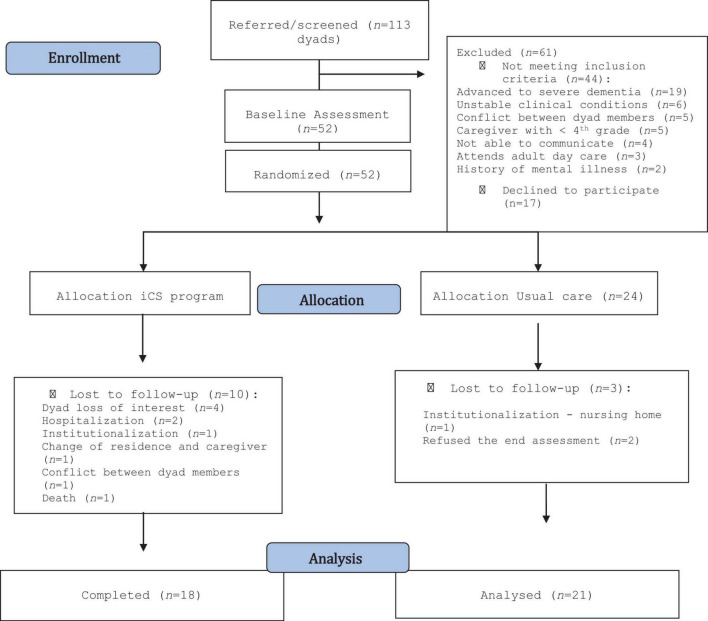
Participant flow through the trial (Moher et al., 2012).

The primary healthcare units of the Regional Health Administration of Northern Portugal referred 113 dyads. Of these, 61 dyads (53.9%) were excluded mostly for not meeting the inclusion criteria (see Figure 1). Thus, 52 dyads were randomized: 28 were allocated to the MD3-P group and 24 to the UC group. Figure 1 shows the number of dropouts and completers in each arm.

The baseline characteristics of the participants who did not complete the study (*n* = 13 dyads) were compared to those of the participants who completed it (*n* = 39 dyads) through the Mann-Whitney *U*-test. None of the sociodemographic characteristics and respective outcomes showed significant differences (*p* > 0.05).

At the end of the intervention, the overall attrition rate was 25.0% (*n* = 13), falling to 23.1% (*n* = 12) if death was not considered. Attrition rates were 12.5% in the UC group (*n* = 3) and 35.7% in the MD3-P group (*n* = 10), decreasing to 32.1% (*n* = 9) if death was excluded. Figure 1 shows the reasons for the losses.

The baseline characteristics of the participants who dropped out (*n* = 13 dyads) were compared to those of the participants who completed the study (*n* = 39 dyads) to assess whether sociodemographic characteristics (e.g., age, gender, education level) or clinical outcomes (e.g., cognitive status or mood) influenced participants’ intention to complete or drop out of the study (Tables 1, 2).

**TABLE 1:** Sociodemographic characteristics of PwCI and caregiver by lost and completed.

	PwCI			Caregiver		
	Completed (*n* = 39)	Lost (*n* = 13)	*p-*value	Completed (*n* = 39)	Lost (*n* = 13)	*p-*value
Age (years), Mean (± SD) Range	79.28 (± 9.67) 60–97	78.77 (± 6.43) 64–90	>0.05	51.54 (± 15.78) 20–82	53.53 (± 17.05) 26–79	>0.05
Women, *n* (%) Men, *n* (%)	28 (71.8) 11 (28.2)	9 (69.2) 3 (30.8)	>0.05	32 (82.1) 7 (17.9)	9 (69.2) 4 (30.8)	>0.05
Married, *n* (%)	27 (69.2)	8 (61.5)	>0.05	30 (76.9)	10 (76.9)	>0.05
Widowed, *n* (%)	12 (30.8)	5 (38.5)		1 (2.6)	–	
Divorced, *n* (%)	–	–		2 (5.1)	2 (15.4)	
Not married, *n* (%)	–	–		6 (15.4)	1 (7.7)	
Education level, Mean (± SD) Range	4.01 (± 2.60) 0–15	3.97 (± 3.20) 1–12	>0.05	8.05 (± 3.84) 4–17	7.4 (± 4.70) 4–15	>0.05
6-CIT, Mean (± SD) Range	13.53 (± 5.69) 4–20	13.46 (± 5.46) 6–20	>0.05	–	–	–
Degree of CI mild, *n* (%) Moderate, *n* (%)	23 (59.0) 16 (41.0)	7 (58.8) 6 (46.2)	>0.05	–	–	–
Son or daughter, *n* (%)	–	–	–	23 (59.0)	5 (38.5)	>0.05
Spouse, *n* (%)				9 (23.1)	5 (38.5)	
Granddaughter, *n* (%)				3 (7.7)	1 (7.7)	
Formal caregiver, *n* (%)				2 (5.1)	2 (15.4)	
Daughter in law, *n* (%)				2 (5.1)	1 (7.7)	
Main caregiver, *n* (%)	–	–	–	24 (61.5)	9 (69.2)	>0.05
Cohabitation, *n* (%)	–	–	–	27 (69.2)	7 (53.8)	>0.05

*MD3-P, intervention group (Making a Difference 3—European Portuguese version); PwCI, Person with Cognitive Impairment; UC, control group (usual care); SD, standard deviation; 6-CIT, 6-item Cognitive Impairment Test.*

**TABLE 2:** Clinical outcomes of PwCI and caregiver by lost and completed.

Outcomes	PwCI			Caregiver		
	Completed (*n* = 39) Mean (± SD)	Lost (*n* = 13) Mean (± SD)	*p-*value	Completed (*n* = 39) Mean (± SD)	Lost (*n* = 13) Mean (± SD)	*p-*value
ADAS-Cog	19.43 (± 7.61)	20.69 (± 7.68)	>0.05	–	–	–
QoL-AD	26.41 (± 5.53)	24.64 (± 6.11)	>0.05	–	–	–
NPI	12.87 (± 11.15)	11.84 (± 13.72)	>0.05	–	–	–
GDS	5.9 (± 3.42)	7.46 (± 4.50)	>0.05	–	–	–
QCPR	56.13 (± 7.40)	55.15 (± 7.06)	>0.05	54.00 (± 9.94)	55.00 (± 7.55)	>0.05
SF12_Mental_	–	–	–	53.33 (± 15.24)	46.45 (± 19.50)	>0.05
SF12_Physical_	–	–	–	55.79 (± 17.17)	55.17 (± 17.77)	>0.05

*ADAS-Cog, Alzheimer’s Disease Assessment Scale; GDS, Geriatric Depression Scale; NPI, Neuropsychiatric Inventory; PwCI, Person with cognitive impairment; QCPR, Quality of the Carer–Patient Relationship (QCPR) scale; QoL-AD, Quality of Life Scale—Alzheimer’s Disease; SD, standard deviation; SF-12, Short Form-12 Health Survey; UC, control group (usual care).*

None of the sociodemographic characteristics or respective clinical outcomes showed statistically significant differences (*p* > 0.05), confirming that the participants’ characteristics did not influence the intention to drop out of the study.

### Sociodemographic Characteristics

Table 3 shows the sociodemographic characteristics of the PwCIs and their caregivers by group (MD3-P and UC) and the baseline assessment results.

**TABLE 3:** Sociodemographic characteristics of PwCI and caregiver.

	PwCI			Caregiver		
	MD3-P (*n* = 28)	UC (*n* = 24)	*p-*value	MD3-P (*n* = 28)	UC (*n* = 24)	*p-*value
Age (years), Mean (± SD) Range	79.5 (± 8.80) 60–97	78.75 (± 9.32) 60–93	>0.05	53.5 (± 15.69) 20–79	60.58 (± 14.88) 20–82	<0.05
Women, *n* (%) Men, *n* (%)	21 (75.0) 7 (25.0)	16 (66.7) 8 (33.3)	>0.05	24 (85.7) 4 (14.3)	17 (70.8) 7 (29.2)	>0.05
Married, *n* (%)	16 (57.1)	19 (79.2)	>0.05	19 (67.9)	21 (87.5)	>0.05
Widowed, *n* (%)	12 (42.9)	5 (20.8)		1 (3.6)	0 (0)	
Divorced, *n* (%)	–	–		3 (10.7)	1 (4.2)	
Not married, *n* (%)	–	–		5 (17.9)	2 (8.3)	
Education level, Mean (± SD) Range	4.07 (± 2.50) 0–15	4.04 (± 2.46) 0–9	>0.05	8.53 (± 4.14) 4–17	7.17 (± 4.14) 4–16	>0.05
6-CIT, Mean (± SD Md Range	13.68 (± 5.72) 6–20	13.33 (± 5.55) 4–20	>0.05	–	–	–
Degree of CI Mild, *n* (%) Moderate, *n* (%)	16 (57.1) 12 (42.7)	14 (58.3) 10 (41.7)	>0.05	–	–	–
Son or daughter, *n* (%)	–	–	–	15 (53.6)	13 (54.2)	>0.05
Spouse, *n* (%)				6 (21.5)	8 (33.3)	
Granddaughter, *n* (%)				2 (7.1)	2 (8.3)	
Formal caregiver, *n* (%)				4 (14.3)	0 (0)	
Daughter-in-law, *n* (%)				1 (3.6)	1 (4.2)	
Main caregiver, *n* (%)	–	–	–	18 (64.3)	15 (62.5)	>0.05
Cohabitation, *n* (%)	–	–	–	16 (57.1)	18 (75)	>0.05

*MD3-P, intervention group (Making a Difference 3—European Portuguese version); Md, Median; PwCI, Person with Cognitive Impairment; UC, control group (usual care); SD, standard deviation; 6-CIT, 6-item Cognitive Impairment Test.*

The randomization process ensured that both groups (MD3-P vs. UC) were similar in terms of sex and degree of cognitive impairment. The groups were also similar in terms of the other sociodemographic variables (age, education level, marital status, *p* > 0.05).

The analysis of differences in the sociodemographic variables of caregivers by group, using the Mann-Whitney U and Chi-Square tests, showed no significant differences (*p* > 0.05), except for age which was higher in the control group [Mean MD3 = 53.5 (± 15.69); Mean UC = 60.58 (± 14.88); U = 229.00; *p* = 0.049].

#### Impact of the Intervention on Persons With Cognitive Impairment

Pre-intervention assessment (ADAS-Cog, QoL-AD, NPI, GDS, QCPR) found no significant differences between groups, except for the quality of life outcome [Mean MD3-P = 28.90 (± 5.27); Md = 26.66; Mean UC = 4.56 (± 5.38); Md = 23.83]. The scores in the QoL-AD scale were higher in the MD3-P group than in the UC group (U = 224.50; *p* < 0.05).

Table 4 shows the mean outcome scores and the total mean difference obtained in the pre- and post-intervention assessments (M_pos–Int_—M_pre–Int_) for each group and each outcome.

**TABLE 4:** Mean outcome scores for MD3-P and UC groups and total pre- and post-intervention mean differences for PwCI.

Outcomes (PwCI)	Pre-intervention assessment		Post-intervention assessment		Mpost-Int—Mpre-Int		
	MD3 –P (*n* = 28) Mean (± *SD*)	UC (*n* = 24) Mean (± *SD*)	MD3-P (*n* = 28) Mean (± SD)	UC (*n* = 24) Mean (± *SD*)	Total (*n* = 52) MD (± *SD*) CI	*p-*value	*d*_cohen_
ADAS-Cog	18.88 (7.11)	19.12 (9.39)	17.94 (8.53)	21.69 (9.73)	0.80 (± 6.20) –0.92–2.53	<0.05	0.651
QoL-AD	28.90 (5.27)	24.56 (5.38)	31.43 (4.13)	25.44 (5.96)	1.87 (± 4.46) 0.61–3.59	>0.05	0.510
NPI	15.88 (13.84)	10.56 (8.25)	9.71 (10.07)	10.37 (8.52)	–1.79 (± 1.78) –5.08–1.48	>0.05	0.320
GDS	5.29 (3.46)	6.37 (3.30)	4.88 (3.69)	5.50 (3.44)	–0.83 (± 3.57) –1.83–0.18	>0.05	0.011
QCPR	59.88 (6.61)	52.50 (7.37)	57.65 (5.96)	52.31 (8.43)	–1.16 (± 6.01) –2.84–0.51	>0.05	0.082

*ADAS-Cog, Alzheimer’s Disease Assessment Scale; CI, 95% confidence interval of the mean of the difference; MD, mean difference; MD3-P, intervention group (Making a Difference 3—European Portuguese version); GDS, Geriatric Depression Scale; NPI, Neuropsychiatric Inventory; Md, median; PwCI, Person with cognitive impairment; QCPR, Quality of the Carer–Patient Relationship (QCPR) scale; QoL-AD, Quality of Life Scale—Alzheimer’s Disease; UC, control group (usual care); SD, standard deviation.*

ADAS-Cog scores in the MD3-P group increased by more than one point from pre- to post-intervention and decreased by more than three points in the UC group, with the difference between the statistically significant (U = 214.50; *p* = 0.02).

Concerning the ES of the MD3-P for cognition, the results suggest that the program had a medium to large ES (d_cohen_ = 0.651). Five PwCIs needed to be treated (NTT) to obtain gains in cognitive performance (in contrast to the control group).

In the ITT analysis, the results on the assessment of the cognitive domains using ADAS-Cog revealed a significant improvement in older adults’ cognition. More specifically, the MD3-P had a more significant positive impact on following commands (U = 103.50; *p* = 0.015) and orientation (U = 89.00; *p* = 0.004). A large ES was found in the orientation domain (d_Cohen_ = 0.88) and a medium ES was found in following commands (d_Cohen_ = 0.75).

The total QoL-AD score (U = 239.00; *p* = 0.07) revealed a marginally significant value for the PwCIs’ quality of life. The mean difference in both groups revealed that the UC group increased its score by one point and that the MD3-P group increased its score by more than two points [MD = 2.60 (± 4.22); Md = 2.67]. The MD3-P intervention had a medium ES on quality of life (d_cohen_ = 0.51). These results show that six PwCIs need to be treated (NTT) for one patient to improve his or her quality of life.

Although NPS improved slightly in the MD3-P group (see Table 4), the differences between groups were not significant (U = 274.00; *p* = 0.25). Depressive symptoms, assessed by GDS-15, improved in both groups, with the UC group showing a greater improvement, although not significant (U = 322.00; *p* = 0.97; d_cohen_ = 0.01). According to the PwCIs version’s QCPR, the quality of the caregiver-PwCI relationship, improved in both groups, being slightly better in the MD3-P group. However, the differences were not statistically significant (U = 297.50; *p* = 0.48; d_cohen_ = 0.082).

#### Impact of the Intervention on the Caregiver

The Mann-Whitney *U*-test revealed no significant differences in the health status scores (SF-12) at baseline between both groups (MD3-P vs. UC). In the QCPR scale—caregiver’s version, the scores were significantly higher in the MD3-P group than in the UC group (*U* = 210.00; *p* < 0.05). Thus, caregivers in the MD3-P group had a better relationship before the intervention than those in the UC group.

Table 5 shows the results of the outcomes evaluated by the caregivers and the total mean differences (M_post–Int_—M_pre–Int_) by group and outcome.

**TABLE 5:** Mean outcome scores for the MD3-P group and total pre- and post-intervention mean differences for caregiver.

Outcomes (caregiver)	Pre-intervention assessment		Post-intervention assessment		Mpost-Int—Mpre-Int		
	MD3-P (*n* = 28) Mean (± *SD*)	UC (*n* = 24) Mean (± *SD*)	MD3-P (*n* = 28) Mean (± *SD*)	UC (*n* = 24) Mean (± *SD*)	Total (*n* = 52) MD (± *SD*) CI	*p*-value	*d*_Cohen_
SF12_Mental_	53.57 (± 16.37)	50.40 (± 17.19)	66.39 (± 8.33)	52.08 (± 12.35)	6.04 (± 15.80) 1.64–10.44	>0.05	0.437
SF12_Physical_	58.00 (± 17.35)	52.88 (± 16.84)	67.50 (± 10.78)	56.88 (± 15.49)	6.96 (± 16.19) 2.45–11.47	>0.05	0.353
QCPR	57.76 (± 10.16)	49.81 (± 9.03)	58.41 (± 8.43)	49.56 (± 9.88)	–0.25 (± 5.36) –1.74–1.24	>0.05	0.27

*CI, 95% confidence interval of the mean of the difference; MD, mean difference; MD3-P, intervention group (Making a Difference 3—European Portuguese version); QCPR, Quality of the Carer–Patient Relationship (QCPR) scale; SD, standard deviation; SF-12, 12-Item Short Form Health Survey; UC, control group (usual care).*

The analysis revealed no statistically significant differences in the outcomes between groups.

### Adverse Events

During the study, some adverse events occurred: (i) one death in the MD3-P group; (ii) a participant in the UC group was institutionalized due to the worsening of psycho-behavioral symptoms; (iii) three participants were hospitalized, two of whom due to worsening of the PwCIs’ health status, and the other due to a fall (one in the MD3-P group; and two in the UC group). These adverse events were not associated with this study. However, immediately after starting the iCS sessions, one caregiver noticed that the family member showed depressive symptoms. This event may have been associated with the intervention because it may have increased the PwCI’s awareness of difficulties in performing the activities. Due to the PwCI’s lack of interest, this dyad dropped out of the study. Three more dyads lost interest in the intervention but provided no justification (see Figure 1).

### Making a Difference 3 Feasibility and Meaningfulness

The attrition rate is an indicator of the acceptability of the iCS program (MD3-P). The attrition rate in the MD3-P group was almost three times higher than that in the UC group [32% (*n* = 9) vs. 12.5 (*n* = 3)]. The main reason reported by the participants for abandoning the program was the loss of interest (30.77%).

Over the 12 weeks, participants had two to 36 sessions. The dyads who completed the study (*n* = 18) had, on average, three weekly sessions (44.4%), two weekly sessions (38.9%), and one and a half weekly sessions (16.7%). Of the eligible participants who did not complete the study, seven had one to 10 sessions, two had 10–20 sessions, and one (one of the hospitalized participants) had 20–30 sessions.

The dyads’ level of satisfaction with the MD3-P sessions was also assessed, ranging from satisfied to very satisfied, and no session was expressively less appreciated.

#### Interviews

Interviews (*n* = 4) were conducted to explore the meaningfulness attributed by the dyads to the iCS program (MD3-P) and their perception of their relationship. The content analysis of the interviews showed the dyads’ opinions about the MD3-P and its impact on their relationship and daily life.

The dyad members recognized that, at an early stage, they did not have high expectations about the MD3-P. However, as the program evolved, the caregivers reported that the PwCIs had improved their spontaneous speech, interaction skills, mood, and were more willing to socialize. Caregivers also recognized that the training helped them implement specific strategies to promote cognitive stimulation in their everyday lives and improve their relationship quality.

“… *This is very important, he’s a lot better*… *no doubt, my father is a more active person now” [Caregiver_1]* “… *she always did the exercises, I think she was engaged during those moments, and she took them seriously “[Caregiver_2]*

The PwCIs recognized that participation in this study was beneficial, regretting that no more sessions were available after the program ended. One participant was not satisfied with the degree of complexity of some sessions but recognized the need for different levels of difficulty. Both interviewed PwCIs reported that, although their cognitive performance had not improved, their mood had improved, and they were more willing to leave the house and socialize.

*“It was good, I had the company of my granddaughter*…, *I made her lunch, and then, we did the exercises” [PcPNC_1]*

*“I enjoyed the exercises, I don’t think I always answered correctly, but I did not feel overwhelmed” [PcPNC_2]*

## Discussion

This study aimed to assess the effectiveness of the European Portuguese version of the iCS program—MD3, and explore the feasibility and meaningfulness attributed to it by PwCIs and their caregivers. As in similar studies, participants who completed the iCS intervention had greater improvements than those who received UC (Quayagen et al., 1995; Orgeta et al., 2015).

With a sample of 52 dyads, the attrition rate was 23.1%, which is justified by the dyads’ loss of interest (30.8%) in the sessions. However, these values are in line with those found in previous studies (Orgeta et al., 2015; Yates, 2016). Of the dyads who completed the study, 44.4% had an average of three weekly sessions. These attrition and low adherence rates can also be explained by the dyads’ low level of knowledge about cognitive impairment and its evolution, lack of information about CS interventions and their role as stabilizers of cognitive disease, and also the caregivers’ burden and low education level (Silva et al., 2020).

Nonetheless, this study revealed significant cognitive improvements in older adults. After the intervention, the PwCIs had improved their cognitive function, namely their orientation and ability to follow commands. The positive effect of the MD3-P on cognition is consistent with previous studies on iCS (Quayagen et al., 1995; Onder et al., 2005). These results suggest that participants may have responded positively to the intervention because they were under-stimulated. Many of their cognitive skills could be preserved but were only minimally manifested due to lack of social stimulus, occupation, and involvement in decision-making. Thus, a pleasant and meaningful interaction with the caregiver during the intervention may have triggered a positive response, translating into health gains (Quayagen et al., 1995; Onder et al., 2005; Valenzuela and Sachdev, 2005).

MCI’s response to intervention should also be explored because older adults with this condition may be more sensitive to an individualized intervention, unlike those with more severe dementia (Mierlo et al., 2010). In this study, about 57% of the participants in the intervention group had MCI or mild dementia, which can explain the more positive response to the intervention. Concerning neuroplasticity and neurogenesis, current evidence suggests that the lower the cognitive damage, the greater the neuroplastic capacity (brain adaptation) and the ability to learn and promote neurogenesis (Valenzuela and Sachdev, 2005; Livingston et al., 2017). The results of this study are promising, so the authors recommend the implementation of cognitive interventions at the earliest stages of cognitive impairment (Livingston et al., 2017). Nevertheless, older adults with more severe cognitive impairment require more differentiated interventions, which may explain the low effectiveness of iCS programs delivered by caregivers to older adults with severe cognitive impairment (Silva et al., 2020).

Unlike the study by Yates (2016), this study found no statistically significant changes in the quality of the relationship between the dyad members. However, qualitative data from the interviews indicate that the relationship improved after the study. Finally, although the implementation of the iCS could have worsened the caregivers’ burden, it had no significant impact on their physical and mental health, which is consistent with Yates (2016). In fact, qualitative data revealed that the caregivers were satisfied with their contribution to their family members’ well-being.

The data obtained for the dyads who completed the 12-week program, showed that the caregivers understood that intellectual activities are vital for the PwCIs’ well-being. Thus, a positive perception of the intervention promotes greater adherence. These results highlight the good feasibility of the MD3-P program, with attrition and adherence rates similar to previous studies on iCS (Orgeta et al., 2015; Yates, 2016).

Both pharmacological and non-pharmacological interventions must take into account the characteristics of their target group (Mierlo et al., 2010). There is no single intervention for all cases, and proper interventions must be designed for each older adult (Mierlo et al., 2010). A tailored intervention can be very effective but only if it is significant enough for its users. Therefore, a significant increase in the number of frail older adults with cognitive impairment requires societal resources/responses and quality services, but above all more differentiated and tailored evidence-based care programs (Prince et al., 2016; Alzheimer’s Disease International [ADI], 2018; Apóstolo et al., 2018).

### Strengths of the Study

The pre-intervention groups were homogeneous, except for the PwCI’s quality of life and the caregivers’ age. Caregivers were significantly younger in the intervention group than in the control group. Before the intervention, caregivers in the iCS program group perceived a better relationship quality than those in the UC group.

The caregivers’ mean age in this study was lower than that found in international studies (Silva et al., 2020). Involving younger caregivers in the delivery of iCS sessions may contribute to the program’s success, given that younger adults tend to have a better understanding of the PwCIs’ difficulties, differentiated skills to conduct CS activities, and better health literacy (Silva et al., 2020).

Blinded outcome assessment was used in this RCT. Dyads from both groups were instructed not to give the assessor any indication of the group to which they were allocated during the study. Another strength of this study was the use of translated and culturally adapted instruments with robust psychometric properties that had already been used in similar research studies (Moniz-Cook et al., 1998; Yates, 2016).

### Limitations of the Study

Although experimental studies allow the identification of causal relationships, they are not exempt from bias. This study should be replicated involving a larger and more diversified sample.

Treatment and control group participants had similar sociodemographic and clinical characteristics at baseline, except for the PwCIs’ quality of life and the caregivers’ age. These differences between groups may threaten the study’s internal validity. Other limitations of this study include its non-representative sample and the high attrition rate. Except for Yates’s study (2016), most of the previous studies focused on iCS, had small samples, justifying it with the complexity of conducting an RCT and the human and economic resources required (Davis et al., 2001; Quayhagen and Quayhagen, 2001; Milders et al., 2013). Although statistical inference is compromised due to the non-representative sample, the magnitude of the intervention effect confirms the clinical importance of these results. Another limitation is the lack of a long-term follow-up assessment. A few studies have found positive long-term effects in similar interventions, which have implications for clinical practice (Silva et al., 2018). Therefore, future studies should address the long-term assessment of the effects of iCS.

Despite these limitations, the methodological design (RCT), the blinded randomization process, the existence of a control group, and the blinded pre- and post-intervention outcome assessments strengthen the study’s internal validity.

## Conclusion

Providing conditions that promote healthy aging in community settings is a social priority. This study focused on individual cognitive stimulation (iCS) as a home intervention conducted by the caregiver for older adults with cognitive deterioration. Three-month intervention was implemented using the European Portuguese version of the iCS therapy program—MD3-P. The intervention was feasible and well accepted by a considerable proportion of older adults and caregivers and produced immediate cognitive benefits at reasonable cost (i.e., two training home visits plus continuous telephone support). This preliminary data extends the benefits of the MD3 to non-English speaking people, giving further support to the value of this therapy program as an innovative and promising instrument in the context of aging in place. Future studies should explore the characteristics of the target population who will benefit most from this type of intervention. This study suggests that the successful implementation and adherence to the MD3-P program require a set of conditions, such as the existence of a good carer-patient relationship, the caregiver’s availability, reasonable levels of health literacy, and the diagnosis of MCI. Hence, the studies should explore these conditions, given their implications for practice.

## Data Availability Statement

The raw data supporting the conclusions of this article will be made available by the authors, without undue reservation.

## Ethics Statement

The studies involving human participants were reviewed and approved by the Ethics Committee of the Regional Health Administration of Northern Portugal (number 27/2017). The patients/participants provided their written informed consent to participate in this study.

## Author Contributions

RS, PS-C, and JA: conceptualization. RS and JA: methodology and funding acquisition. RS and EB-C: formal analysis. RS, EB-C, PS-C, and ARC: investigation and data curation. RS: writing—original draft preparation. RS, EB-C, PS-C, ARC, and JA: writing—review and editing. JA: supervision and project administration. All authors have read and agreed on the published version of the manuscript.

## Conflict of Interest

The authors declare that the research was conducted in the absence of any commercial or financial relationships that could be construed as a potential conflict of interest.

## Publisher’s Note

All claims expressed in this article are solely those of the authors and do not necessarily represent those of their affiliated organizations, or those of the publisher, the editors and the reviewers. Any product that may be evaluated in this article, or claim that may be made by its manufacturer, is not guaranteed or endorsed by the publisher.

## Footnotes

1
http://www.random.org
